# Color needs luminance for visual selection during scene search

**DOI:** 10.3758/s13414-026-03226-7

**Published:** 2026-03-06

**Authors:** Anke Cajar, Jochen Laubrock

**Affiliations:** Department of Psychology, University of Potsdam, Karl-Liebknecht-Str. 24-25, 14476 Potsdam, Germany

**Keywords:** Visual search, Scene perception, Eye tracking, Gaze-contingent displays, Color perception, Luminance perception

## Abstract

When searching visual scenes, we use low-level visual information from objects’ defining features such as color and luminance contrasts. What is the relative influence of color and luminance for saccade target selection? Basic perceptual research suggests that we are not very sensitive to peripheral color, yet color is thought to be an important basic feature guiding visual search. Previous gaze-contingent research shows that targets can be localized faster in color than in grayscale scenes, therefore the availability of color in the visual periphery indeed helps visual search. However, object boundaries are typically defined by both color and luminance contrasts. Here we study the isolated roles of color and luminance during object-in-scene search by presenting either color-only or luminance-only contrasts in peripheral vision, using a gaze-contingent moving-window display with three varying window sizes. We found that peripheral target selection and search performance were more efficient with luminance contrasts, whereas color was used only sparingly beyond the parafovea. We conclude that color contrasts in peripheral vision are only efficiently used in scene search when they are jointly occurring with luminance contrasts.

When we look at a color photograph, we have the impression of a colorful scene, with color everywhere in the picture. However, basic perceptual research indicates that the use of peripheral color for visual search is severely limited. In contrast, results from the visual search tradition suggest that color is a basic feature guiding search. How can these results be reconciled? Here, we investigate the contribution of color to search efficiency in real-world scenes. We restricted the extent of color and luminance previews per fixation. Before detailing our research setup, we briefly review existing research on the use of color and luminance in visual perception and search.

## Luminance and color contrasts in vision

A fundamental goal of vision is object recognition, which is aided by both luminance and color contrasts. Color and luminance are processed in partial independence. For cone vision, contrast sensitivity relies on opponent processes arising from three types of cones (S, M, L), coding for, roughly, red vs. green (L-M) and blue vs. yellow (S-(L+M)) color contrasts, and one additive mechanism coding for luminance (L+M). Rod vision is generally insensitive to color and only sensitive to luminance. How color and luminance act together in object segmentation is far from solved (Fine, MacLeod, & Boynton, 2003; Hansen & Gegenfurtner, 2009; Hansen & Gegenfurtner, 2017). Intuitively, they co-occur at object boundaries, and one or the other may be more important depending on illumination and accidental viewing conditions. Saliency models (Itti & Koch, 2000) explicitly represent this idea by assuming independent feature maps, which are combined to define an object. There is some empirical support that color and luminance contrasts combine to bias saccade target selection (Engmann et al., 2009).

What is the relative contribution of color and luminance contrasts to perception? This question can be studied by comparing grayscale with chromatic isoluminant stimuli, from which color and luminance contrasts have been removed, respectively. At a basic level, the detection of isoluminant color patterns is facilitated by subthreshold luminance patterns, whereas color does not facilitate luminance detection (Switkes, Bradley, & De Valois, 1988), suggesting that luminance gates color signals into the cortex (Shapley, 1990). This is consistent with the L+M luminance signal traveling along the faster magnocellular pathway, whereas opponent L-M and S-(L+M) color channels use the slower parvo- and koniocellular pathways, respectively (Dobkins, 2000; Gegenfurtner & Kiper, 2003). Turning stimuli isoluminant mainly affects the L- and M-cones, because their absorption spectra overlap almost completely.

Previous work investigating the individual contributions of chromatic and achromatic signals with simple stimuli suggests that shape discrimination is better with luminance than with color contrasts (Mullen & Beaudot, 2002), and that abrupt luminance changes pop out, whereas abrupt color changes do not, suggesting preattentive parallel processing of luminance, but not color signals (Theeuwes, 1995). Furthermore, rapid scene categorization has been shown to rely on luminance information, but not on color cues (Delorme, Richard, & Fabre-Thorpe, 2000). However, in the real world, luminance and color contrasts might coincide at object boundaries. Color edges are better indicators of what observers see as the important object contours in a scene, when viewing these scenes for a long time (Hansen & Gegenfurtner, 2017). When color changes, it is a new object; when luminance changes, it could be a new object, but it could also be a shadow. Evidence suggests that preattentive processing of visual stimuli is facilitated when stimuli contain both luminance and color signals, compared with luminance signals alone (Hardman, Töllner, & Martinovic, 2020). Both color and luminance are also necessary for unimpaired object recognition (Poth & Schneider, 2016).

## Color vs. luminance in foveal and peripheral vision

How does sensitivity to luminance or color contrasts change across the visual field? Cone spacing as well as the rod-to-cone ratio increase with retinal eccentricity, therefore spatial acuity decreases (Mullen & Kingdom, 2002; Witzel & Gegenfurtner, 2014).

Foveally, overall contrast sensitivity is highest for medium-frequency gratings of about 3–7 cpd. This bandpass characteristic is due to integration over a number of channels centered at different spatial frequencies (Campbell & Robson, 1968). Sensitivity for luminance contrasts decreases with increasing eccentricity. The gradient is very steep at high spatial frequencies but considerably shallower at low spatial frequencies. Both the maximum sensitivity and the spatial frequency at which it occurs decrease in the periphery (Hilz & Cavonius, 1974).

The spatial resolution for color in the periphery decreases even more steeply than for luminance, with red-green sensitivity dropping even more steeply than blue-yellow sensitivity (Mullen & Kingdom, 2002; Mullen, Sakurai, & Chu, 2005).

Because of this drop in acuity for basic features like luminance and color, object perception is also impaired. Object selection in the visual periphery is based on broad, low-frequency blobs rather than fine detail. Even below the object level, there are peripheral shape metamers which make large disruptions of the peripheral visual field invisible (Freeman & Simoncelli, 2011)

## Peripheral and foveal vision in visual search

Because peripheral acuity is so low, we constantly need to move our eyes to bring objects of interest into foveal vision. When searching visual scenes, we solve two ongoing tasks in parallel. We inspect the fixated object using high-acuity foveal vision, while simultaneously selecting the next object blob of interest using peripheral vision, to which we then execute a saccade to bring it into foveal vision for closer inspection. Although the fovea is small, foveal inspection tends to dominate fixation duration, because it usually takes longer than peripheral selection (Laubrock, Cajar, & Engbert, 2013). Objects have been shown to play an important role in guiding attention in scenes (Nuthmann & Henderson, 2010; Stoll, Thrun, Nuthmann, & Einhäuser, 2015). Thus, low-frequency peripheral vision must contain sufficient information for object segmentation.

## Color in visual search

Given the perceptual results, one might expect color to be relatively inefficient in guiding peripheral selection. However, color is considered one of the few basic features in visual search that produce a pop-out effect (Treisman & Gelade, 1980; Wolfe, 2021). Unlike for many other features, search times for a uniquely colored item among a set of distractors do not increase with the number of distractors. Color is thus considered a basic preattentive feature. Basic features attract attention automatically, in a bottom-up fashion, whereas top-down attention involves a higher-level control system that can weigh different basic and higher-level features according to task demands (Itti & Koch, 2000).

## Color in real-world scenes

Are color contrasts important for delineating object boundaries in real-world scenes? Most studies on the effects of color removal in real-world scenes removed color from the entire scene (Cajar, Engbert, & Laubrock, 2020; Einhäuser, Atzert, & Nuthmann, 2020; Ho-Phuoc, Guyader, Landragin, & Guérin-Dugué, 2012; Hwang, Higgins, & Pomplun, 2007), with only a few studies removing color gaze-contingently in either central or peripheral vision (Cohen, Botch, & Robertson, 2020; Nuthmann & Malcolm, 2016). Studies consistently show longer search times and lower search accuracy when removing color while searching objects or scene patches (Cajar et al., 2020; Hwang et al., 2007; Nuthmann & Malcolm, 2016). Additionally, shorter saccade amplitudes and longer scanning times were observed (Cajar et al., 2020; Nuthmann & Malcolm, 2016), indicating that color is an important feature for peripheral target selection and object localization in scene search; Hwang et al. (2007) suggest that color might even dominate visual guidance over other features, such as intensity or contrast. Most studies also found longer fixation durations with color removal in scenes during free viewing and search (Einhäuser et al., 2020; Ho-Phuoc et al., 2012; Nuthmann & Malcolm, 2016, see, however, Cajar et al., 2020, who found no effect on fixation durations), and longer object verification (i.e., identification) times (Cajar et al., 2020; Nuthmann & Malcolm, 2016). Taken together, previous results clearly indicate that color aids peripheral object localization as well as central object identification in scenes.

## Disentangling the effects of color and luminance

The isolated role of peripheral color vision in target selection remains unclear. On the one hand, basic perceptual results indicate that color alone is relatively unimportant in the periphery. On the other hand, search times and eye-movement statistics in gaze-contingent scene viewing are affected by a lack of peripheral color, and color and luminance can add linearly to influence fixation locations in scenes (Engmann et al., 2009).

A critical difference between the two approaches is that basic perceptual research has sought to isolate the effects by comparing color and luminance contrasts. Previous gaze-contingent scene studies, however, have always contrasted color with grayscale previews. Color was thus added on top of luminance contrasts already being present, and the independent effect of color without luminance was never evaluated. Thus, while these studies point to color as an important contributor to search efficiency, they do not allow us to clearly disentangle the relative importance of color and luminance for scene viewing. Previous eye-movement studies seem to have implicitly assumed that luminance is more important than color.

The present study directly compares the unique contributions of luminance and color contrasts in the periphery by comparing grayscale with isoluminant chromatic peripheral previews. We monitored participants’ eye movements during an object-in-scene search task in photographs of real-world scenes. Keeping central vision unimpaired, we presented grayscale (color-removed) or isoluminant color (luminance-removed) versions of the scene in peripheral vision using a gaze-contingent moving window (see Nuthmann & Malcolm, 2016, who used a similar setup for peripheral color removal). We varied the size of the window to assess at which eccentricities color or luminance contrasts dominate. Extrapolating from perception experiments, we expected luminance to have a higher weight in the periphery, thus still producing effects for larger window sizes where color might already fail to affect viewing behavior. As in previous research (Cajar, Engbert, & Laubrock, 2016a; Cajar, Schneeweiß, Engbert, & Laubrock, 2016b; Laubrock et al., 2013; Nuthmann, 2014; Nuthmann & Malcolm, 2016) we expected effects on search behavior, saccade amplitudes, and fixation durations.

## Method

### Participants

Thirty-five University of Potsdam students (26 female, nine male, mean age 22.9, range 18–34 years) participated in the experiment. Participants had normal or corrected-to-normal vision and normal color discrimination. They were naive to the purpose of the experiment and received course credit or monetary compensation. Participants gave written informed consent prior to the experiment conforming to the Declaration of Helsinki.

### Apparatus

Stimuli were presented on a VIEWPixx monitor (VPixx Technologies) with a resolution of 1920 $\times$ 1080 pixels and a refresh rate of 120 Hz. Stimulus presentation was controlled with Matlab (The MathWorks, Natick, MA, USA) using the Psychophysics (Brainard, 1997; Kleiner, Brainard, & Pelli, 2007) and EyeLink (Cornelissen, Peters, & Palmer, 2002) Toolboxes. Viewers were seated 70 cm (27.6 inches) away from the monitor with their head stabilized by a head-chin rest. Gaze position of the dominant eye was tracked during binocular viewing with the EyeLink 1000 system (SR Research, Ontario, Canada).

### Stimuli and design

**Fig. 1:**
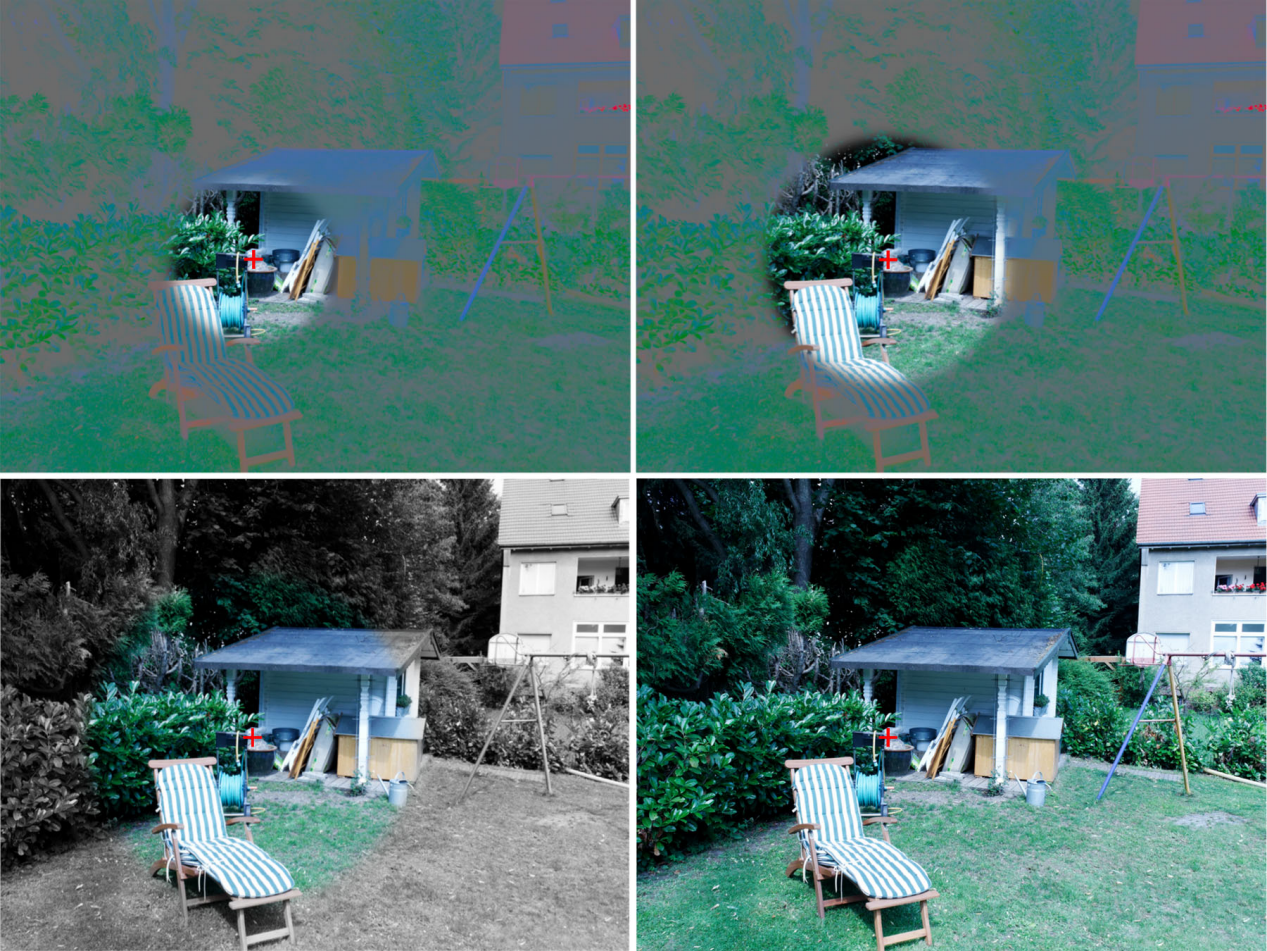
Illustration of three of the six filter conditions and the control condition. The *red cross* indicates the current gaze position. The target object in this example is the gray watering can standing on the lawn. (*Top left*) Luminance removed beyond 4$^\circ$ eccentricity. (*Top right*) Luminance removed beyond 6$^\circ$ eccentricity. (*Bottom left*) Color removed beyond 8$^\circ$ eccentricity. (*Bottom right*) Unfiltered control condition. Note that you need color to appreciate this figure; on a grayscale printout, the masks in the top panels will look homogeneous gray and the bottom panels will look identical

Stimuli were 119 images of real-world scenes from the BOiS database (Mohr et al., 2016) resized to 1400 $\times$ 1050 pixels, subtending a visual angle of 31.6$^\circ$ $\times$ 23.7$^\circ$. Of these images, 100 depicted indoor and 19 depicted outdoor scenes. Each scene showed the target object at a predictable location with respect to scene context (see Fig. 1). Pictures of search targets rather than word cues were presented before each trial. Although the actual target would look slightly different in the filtered periphery, we thus ensured that all participants used the same internal target representation.

For each scene color- and luminance-removed versions were prepared in advance. Color-removed versions were created by converting the color images to grayscale using MATLAB’s rgb2gray function. For luminance-removed versions, we converted images to CIELAB space and set the L value of all pixels to the average luminance of the image. This procedure led to about five-fold measured RMS contrast reduction, which should be sufficient for all practical purposes. Note, however, that whenever we use the term ‘isoluminant‘ in the following, we refer to near-isoluminant stimuli.

For gaze-contingent filtering of the peripheral visual field (i.e., removal of color or luminance), a foreground and a background image were merged in real-time using alpha blending. The foreground was the original scene and the background was the filtered version of the scene. To avoid sharp boundaries, a 2D hyperbolic tangent with a slope of 0.06 served as a blending function for creating the alpha mask. The inflection point of the function corresponded to the radius of the gaze-contingent moving window. The alpha mask was centered on the current gaze position and defined the transparency value.

Two filter types (color-removal, luminance-removal) were crossed with three window sizes (small, medium, large), yielding six filter conditions: luminance removed beyond 4$^\circ$, 6$^\circ$, or 8$^\circ$ eccentricity, and color removed beyond 4$^\circ$, 6$^\circ$, or 8$^\circ$ eccentricity (for example stimuli, see Fig. 1). A control condition without filtering served as a baseline. This yielded 17 trials per condition.

A Latin square design assured counterbalancing of condition–scene assignments across participants. The scenes were presented in random order.

### Procedure

At the beginning of the experiment, and after every 15 trials, a nine-point calibration was performed. At the beginning of each trial, fixation was checked—the viewer’s gaze had to stay within an area of $1.5^\circ \times 1.5^\circ$ around the screen center for 200 ms. After a successful check, the actual trial started; if the check failed three times, a re-calibration was scheduled.

Three example trials familiarized participants with the task and gaze-contingent moving window procedure. Each trial started with a pictorial cue of the target object on a black background presented for two seconds. Subsequently, a central black cross was presented to ensure viewers started exploring in the image center. After the cross was fixated the scene was revealed. Viewers were instructed to find the scene’s target object as quickly as possible and to press the left button of the computer mouse once they found it. There was a response deadline of 60 s.

### Data preparation

Saccades were detected in the time series of gaze positions using a velocity-based algorithm (Engbert & Kliegl, 2003; Engbert & Mergenthaler, 2006) with a relative velocity threshold of 6 standard deviations and a minimum duration of eight data samples. A total of 13 trials (0.31%) were removed due to poor calibration or too much data loss. Three additional trials were removed from search analyses because they reached the response deadline of 60 s. Single fixations and saccades were removed if they were neighboring eye blinks or were outside the scene. If the first or last trial event was an ongoing saccade, it was also removed. In total, 24,667 fixations and 20,971 saccades remained for eye-movement analyses.

### Data analyses

Data were analyzed with R (R Core Team, 2025, version 4.5.1). All dependent measures were analyzed using linear mixed-effects models (LMMs) (Bates, Maechler, Bolker, & Walker, 2015b) assuming random intercepts and slopes for participants and scenes, with selection of random-effects structures following the procedure suggested by Bates, Kliegl, Vasishth, and Baayen (2015a). Fixed-effects parameters representing effects of the experimental manipulations were estimated using six contrasts with effect coding testing for: (1) a difference between the unfiltered control condition and all filter conditions, (2) a difference between filter types (luminance- vs. color-removal), (3) a difference between 4$^\circ$ and 6$^\circ$ windows, (4) a difference between 6$^\circ$ and 8$^\circ$ windows, (5) an interaction of filter type for 4$^\circ$ and 6$^\circ$ windows, and (6) an interaction of filter type for 6$^\circ$ and 8$^\circ$ windows. Each contrast matrix was set up so that conditions expected to yield stronger deviations from the control condition in the respective measure were assigned positive contrast coefficients. Model coefficients and standard errors for a given set of analyses were scaled to an easily reportable order of magnitude; the scaling factor is reported as scale once per set.

For each measure, we also ran post hoc LMMs testing each filter condition against the unfiltered control to evaluate whether information at that eccentricity was used. Statistically significant effects are reported below.

In order to achieve normally distributed residuals for LMMs, we transformed positively skewed dependent measures using the Box-Cox procedure (Box & Cox, 1964), yielding logarithmic transformations for fixation durations, saccade amplitudes, and verification times, inverse transformations for search times and search initiation times, and inverse square-root transformations for scanning times. Signs of contrast coefficients for search times, initiation times and scanning times were inverted for the report to match direction of fixed effects with the other measures. For all measures of search behavior, only trials in which the target was actually fixated were included in the analyses.

## Results

**Fig. 2:**
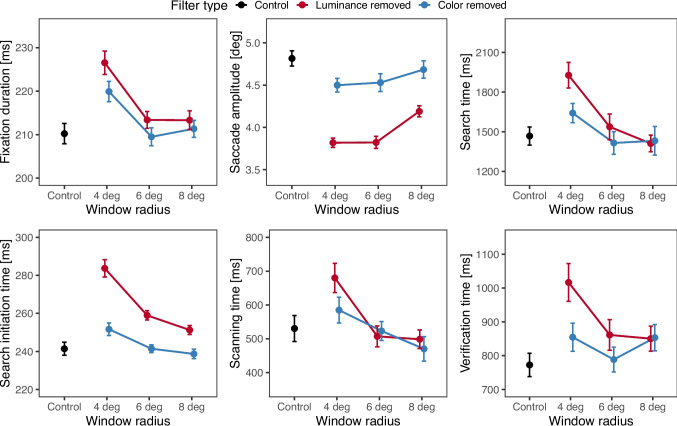
Mean eye-movement and search behavior. *Top row*: Fixation durations, saccade amplitudes, and search time. *Bottom row*: Search initiation time, scanning time, and verification time. *Error bars* represent 95% within-subject standard errors with Cosineau–Morey correction (Cousineau, 2005; Morey, 2008)

Figure 2 illustrates eye-movement and search behavior for all seven conditions of the experiment.

### Fixation durations

Mean fixation duration across conditions was 218 ms ($$\textit{SD} = 102$$ ms). Fixation durations were longer when searching filtered than unfiltered scenes (scale $$ 10^{-2}$$; $$b = 2.67, \textit{SE} = 1.09, t = 2.45, p =.019$$), and longer with luminance removal than with color removal ($$b = 2.50, \textit{SE} = 1.08, t = 2.31, p =.027$$). The greatest effect was the difference between 4$$^\circ $$ and 6$$^\circ $$ windows, with the smaller radius inducing longer fixation durations ($$b = 6.29, \textit{SE} = 1.07, t = 5.89, p <.001$$). Both filter types with 4$$^\circ $$ windows differed from the unfiltered control condition ($$ps <.001$$). The comparison between 6$$^\circ $$ and 8$$^\circ $$ windows and both interactions of filter type and window size were statistically non-significant ($$p =.823$$, $$p =.432$$, and $$p =.520$$, respectively).

### Saccade amplitudes

Mean saccade amplitude across conditions was $$4.4^\circ $$ ($$\textit{SD} = 3.5^\circ $$). In general, the saccade amplitudes decreased when searching filtered scenes (scale $$ 10^{-2}$$; $$b = -8.10, \textit{SE} = 1.65, t = -4.90, p <.001$$). This decrease was stronger with peripheral luminance than with color removal ($$b = -6.34, \textit{SE} = 1.84, t = -3.44, p =.001$$). Surprisingly, there was no difference in saccade amplitudes between 4$$^\circ $$ and 6$$^\circ $$ windows ($$p =.179$$); however, saccades were shorter with 6$$^\circ $$ than with 8$$^\circ $$ windows ($$b = -4.97, \textit{SE} = 1.56, t = -3.19, p =.001$$). There was no interaction between filter type and 4$$^\circ $$ and 6$$^\circ $$ windows ($$p =.955$$), but a significant interaction between filter type and 6$$^\circ $$ and 8$$^\circ $$ windows ($$b = -3.10, \textit{SE} = 1.56, t = -1.99, p =.046$$), indicating a larger difference between filter types with 6$$^\circ $$ windows. Post hoc comparisons with the unfiltered control revealed that luminance removal decreased amplitudes at all window sizes ($$p<.001$$ for 4$$^\circ $$ and 6$$^\circ $$, $$p=.010$$ at 8$$^\circ $$), while color removal had much weaker effects, which were only significant at 4$$^\circ $$ ($$p =.003$$) and marginally significant at 6$$^\circ $$ ($$p =.065$$).

### Search times

Mean search time across conditions was 1549 ms ($\textit{SD} = {2,046}$ ms). Overall, search times were longer with filtered than with unfiltered scenes (scale $10^{-5}$; $b = 7.31, \textit{SE} = 2.01, t = 3.64, p <.001$). Comparing filter types, search took longer with peripheral luminance removal than with color removal ($b = 7.76, \textit{SE} = 1.71, t = 4.54, p <.001$). Expectedly, search times increased with decreasing window radius—they were longer with 4$^\circ$ than 6$^\circ$ ($b = 15.0, \textit{SE} = 1.70, t = 8.81, p <.001$), and longer with 6$^\circ$ than 8$^\circ$ ($b = 4.24, \textit{SE} = 1.39, t = 3.05, p =.003$). An interaction of filter type and 4$^\circ$ and 6$^\circ$ windows occurred ($b = 5.14, \textit{SE} = 1.33, t = 3.86, p <.001$), because luminance removal yielded longer search times than color removal with 4$^\circ$, but not 6$^\circ$ windows. No difference between filter types occurred with 6$^\circ$ vs. 8$^\circ$ windows ($p =.405$). Search-time increase compared with the control condition was only significant at 4$^\circ$ for color removal ($p <.001$), whereas luminance removal had significant effects at 4$^\circ$ and 6$^\circ$ ($p\text {s} <.001$).

### Search epochs

To gain a deeper understanding of the effects of luminance and color removal on search, in a corollary post-hoc analysis we decomposed the search process into three epochs: search initiation time (time between scene onset and the first actively initiated saccade, scanning time (end of search initiation until the first fixation in the target area), reflecting target localization, and verification time (time from the first fixation in the target area until search termination), reflecting target identification (Malcolm & Henderson, 2009). Note that in order to promote natural search behavior, we did not require participants to directly fixate the target once they had found it. We therefore used a data-driven approach to decide when a target had been found, based on gaze location. A criterion of 5.5$^\circ$ was derived from an analysis of search performance as a function of gaze distance from target, it is slightly larger than the 4$^\circ$ often chosen in forced fixation tasks. We defined the first fixation with a maximum distance of 5.5$^\circ$ to the target as the end of scanning time. For 10% of these trials, search initiation time could not be determined because of blinks, glissades, or saccades coinciding with scene onset. Additional trials were excluded from scanning times when the target was located near the initial fixation location, and the target was found within fewer than four fixations. In total, 90.3% of target-found trials were available for evaluation of initiation and verification times, and 50% for scanning times.

#### Search initiation times

Mean search initiation time across conditions was 253 ms ($$\textit{SD} = 70$$ ms). Overall, initiation times were longer with filtered than with unfiltered scenes (scale $$ 10^{-4}$$; $$b = 1.78, \textit{SE} = 0.37, t = 4.78, p <.001$$). Comparing filter types, search initiation took longer with peripheral luminance removal than with color removal ($$b = 3.17, \textit{SE} = 0.32, t = 9.76, p <.001)$$. Additionally, search initiation times scaled with window size, with slower initiation for 4$$^\circ $$ than 6$$^\circ $$ windows ($$b = 2.11, \textit{SE} = 0.37, t = 5.75, p <.001$$) and slower initiation for 6$$^\circ $$ than 8$$^\circ $$ windows ($$b = 1.0, \textit{SE} = 0.33, t = 3.07, p =.002$$). The difference between 4$$^\circ $$ and 6$$^\circ $$ windows was larger for luminance than for color removal, indicated by a significant interaction between filter type and these window sizes ($$b = 0.89, \textit{SE} = 0.35, t = 2.52, p =.013$$). Finally, post hoc LMMs showed a significant increase in search initiation times compared with the unfiltered control condition for all three window sizes when luminance was removed in the periphery ($$p <.001$$ with 4$$^\circ $$ and 6$$^\circ $$ windows, and $$p =.003$$ with 8$$^\circ $$ windows), but only with 4$$^\circ $$ windows when color was removed ($$p = .010$$).

#### Scanning times

Mean scanning time across conditions was 551 ms ($$\textit{SD} = {573}$$ ms). Scanning times were longer with filtered than with unfiltered scenes (scale $$ 10^{-3}$$; $$b = 2.22, \textit{SE} = 0.92, t = 2.42, p =.018$$). In filtered scenes, 4$$^\circ $$ windows involved longer scanning times than 6$$^\circ $$ windows ($$b = 2.63, \textit{SE} = 0.88, t = 2.98, p =.004$$), and 6$$^\circ $$ windows longer scanning times than 8$$^\circ $$ windows ($$b = 1.89, \textit{SE} = 0.82, t = 2.32, p =.020$$). There was no effect of filter type ($$p =.259$$). The interactions of filter type and window size were also non-significant ($$p =.241$$ and $$p =.335$$ respectively). Compared with the control condition, effects of luminance removal and color removal were both significant only with 4$$^\circ $$ windows ($$p <.001$$ and p = .001 respectively), and marginally significant for color at 6$$^\circ $$ ($$p=.076$$).

#### Verification times

Mean verification time across conditions was 852 ms ($\textit{SD} = {1064}$ ms). Verification times did not increase with filtered compared to unfiltered scenes ($p =.159$). Verification times were slightly longer for luminance than for color removal (scale $10^{-2}; b = 6.77, \textit{SE} = 3.16, t = 2.14, p =.038$). Verification times were substantially longer with 4$^\circ$ than with 6$^\circ$ windows ($b = 14.34, \textit{SE} = 3.23, t = 4.44, p <.001$), and this effect was enhanced with luminance compared to color removal ($b = 6.25, \textit{SE} = 2.84, t = 2.20, p =.028$). The 6$^\circ$ and 8$^\circ$ window size contrast was not involved in either a main effect ($p =.933$) or an interaction ($p =.849$). Compared with the control condition, verification times with 4$^\circ$ windows increased significantly when luminance was removed ($p <.001$) and marginally ($p =.089$) when color was removed.

## Discussion

The present study investigated the isolated effects of luminance and color in the visual periphery during object search in real-world scenes. We selectively removed luminance or color contrasts from the scenes at various levels of eccentricity. Removing luminance decreased saccade amplitudes and increased search initiation time for all window sizes tested, increased search times for 4$^\circ$ and 6$^\circ$ windows, and scanning and verification times as well as fixation durations for 4$^\circ$ windows. In contrast, effects of color removal were only found for 4$^\circ$ windows. These results suggest that luminance independent of color affects saccade target selection and some aspects of search behavior at least as far as 8$^\circ$ in the periphery, whereas luminance-independent effects of color on target selection and search are largely restricted to parafoveal viewing. For fixation durations and search behavior the effect of luminance removal was greatest with 4$^\circ$ windows, where the difference between luminance and color was also most pronounced.

Removing color affected all measures at 4$^\circ$ eccentricity, replicating (Nuthmann & Malcolm, 2016), who still found these effects with 5$^\circ$ windows. However, present results show that removing color affected no measure with windows of 6$^\circ$ radius and larger, suggesting that the use of color contrasts for search in natural scenes seems to be limited to foveal and parafoveal vision. These results are in line with findings obtained using resting eyes, showing that sensitivity for color drops off more sharply with eccentricity than sensitivity for luminance contrasts.

Previous work on visual search, as well as on gaze-contingent scene-viewing, also suggests that search times are shorter in color scenes than in grayscale scenes and that low-frequency peripheral color information is critical for facilitating search (Cajar et al., 2020; Hwang et al., 2007; Nuthmann & Malcolm, 2016). However, in all of these studies, luminance and chromaticity were not studied independently, but rather the additional effect of color was evaluated, given existing luminance contrasts. In the present study, we added a condition with pure color contrasts, thereby allowing us to evaluate the relative importance of color and luminance for peripheral target selection.

In the context of earlier research, our results show that color is only beneficial if luminance information is additionally available, and that color *alone* does not guide search in the periphery. Results rather point to an interactive mechanism for peripheral selection, where luminance is mandatory and color can provide additional benefits. The most parsimonious interpretation in the context of earlier findings is that peripheral color is beneficial for search, but mainly if it is supported by luminance. This interpretation is in accord with Hardman et al. (2020), who found that luminance contrasts added to chromaticity contrasts accelerated an event-related EEG component compared to pure luminance contrasts. More generally, it is in line with Shapley’s interpretation that luminance gates color (Shapley, 1990). Color and luminance might be integrated during preattentive processing, but luminance comes first.

Recent research suggests that objects and higher-level features relying on object recognition, such as scene composition, are more important than low-level visual properties in guiding real-world scene search (Çelikkol, Laubrock, & Schlangen, 2023; Henderson & Hayes, 2017; Kümmerer, Bethge, & Wallis, 2022; Nuthmann & Henderson, 2010; Võ, 2021), stressing the importance of late attentional selection. Some authors even argue that early saliency affects attention only indirectly, acting through recognized objects (Einhäuser, Spain, & Perona, 2008).

However, if peripheral vision is low-frequency and objects cannot be fully resolved in the periphery, how can they be selected by attention for further foveal inspection? In context, the present results suggest that color is important for peripheral object localization, but mainly if the objects differ from their surroundings in luminance. If so, there might be a perceptual mechanism akin to a coincidence detector that enhances joint contrasts for later stages of object detection. The present results suggest that such a hypothetical coincidence detector would be primed by the luminance signal.

Luminance and color contrasts are uncorrelated in static images and can independently signal object boundaries (Hansen & Gegenfurtner, 2009). However, this still leaves the possibility that joint color and luminance contrasts are useful, possibly because they might be more likely at object boundaries across lighting and accidental viewing conditions. Despite some evidence that object boundaries coincide with contrasts of both luminance and color (Fine et al., 2003), this issue is far from resolved. The different acuity characteristics of color and luminance channels further suggest that a combination of information at different (but low) spatial frequencies should be considered.

In summary, color contrasts in the absence of luminance contrasts seem to contribute little to the selection of peripheral objects for close inspection during scene viewing. How can this be reconciled with earlier results showing that color facilitates visual search? Earlier studies only added color to luminance, in which case, color indeed helps. The present results suggest that, when assessed independently, luminance is more important for saccade target selection and object search. Taken together, we interpret these findings to suggest that color information aids in peripheral object segmentation and localization mainly after luminance contours have been identified.

## Data Availability

The data are available at https://osf.io/sy92a/. Material was taken from the open-access Berlin Object in Scene Database available at https://info.ni.tu-berlin.de/photodb/download.html.

## Code Availability

The analyses scripts for the experiment are available at https://osf.io/sy92a/.
